# Investigation of auditory thresholds in type 2 diabetic patients compared to non-diabetic cases

**Published:** 2014

**Authors:** Keivan Kiakojouri, Mohsen Monadi, Mahboobeh Sheikhzadeh, Pouria Taghinejad Omran, Mohammad Ali Bayani, Soraya Khafri

**Affiliations:** 1 Department of Otolaryngology, Babol University of Medical Sciences, Babol, Iran.; 2 Department of Audiology and Speech pathology, Babol University of Medical Sciences Babol, Iran.; 3 University of Social Welfare and Rehabilitation Sciences, Tehran, Iran.; 4 Babol University of Medical Sciences, Babol, Iran.; 5 Department of Internal Medicine, Babol University of Medical Sciences, Babol, Iran.; 6 Department of Social Medicine and Health, Babol University of Medical Sciences, Babol, Iran.

**Keywords:** Hearing loss, Audiometry, SRT, SDS, frequency

## Abstract

***Background: ***Hearing loss is one of the common complaints of diabetics. The aim of this study was to evaluate the hearing status of diabetic patients in Babol, North of Iran.

***Methods:*** The hearing status of 50 type 2 diabetic patients (case group) and 50 healthy individuals (control group) were evaluated from October 2011 to September 2012. Audiometry was done with the frequencies of 250, 500, 1000, 2000, 4000, 6000, 8000 HZ and speech tests like SRT (speech reception threshold), SDS (speech discrimination score) were performed. The data were collected and analyzed.

***Results:*** The mean age of case group was 50.1±3 and in control group was 49.9±3.2 years. Hearing thresholds were 10.55.6, 10.76.1, 15.27.1, 169.6, 21.213.5, 26.416.5, 28.32 db in the right ear of the case group and 11.25.3, 9.74.9, 127.1, 14.29.4, 20.913.3, 25.115.6, 27.620.2 db in control group with different frequencies (p<0.05). Similar results were obtained in the left ear of both groups. The mean SRT in the right ear of the case group was 15.77.7 and control group was 9.24.8 and 13.56.9 in case and 9.14 in the left ear of case group (P=0.0001). SDS was 94.33.7 and in 96.23.3 in the right ear for the case and control group, respectively (P=0.0001). For the left ear, these values were 94.44.4 and 95.93.2, respectively (P=0.023).

***Conclusion: ***The results show that hearing loss in different frequencies and speech reception threshold were higher in diabetic group but speech discrimination score was higher in normal individuals. Audiological monitoring is recommended in diabetic patients during therapy.

Diabetes is one of the most important metabolic diseases in the world, and because of its different side effects, has adverse impacts on health and the people’s quality of life. Some of its common side effects are retinopathy, nephropathy and vasculapathy. Besides, hearing loss is one of its chronic side effects (1). Although this disease does not have any definite treatment, but we can prevent from its demonstrations. The relationship between diabetes and hearing impairment has been argued for more than one century and yet, there is not any agreement about this matter (2-3). For the first time, Jordao introduced a diabetic patient who had hearing loss in 1875 (4). Since then many studies have been conducted Some of them acknowledged and other studies rejected the relationship between hearing loss and diabetes (5). To explain the relationship between diabetes and hearing loss, some researchers proposed angiopathic origin and others proposed neurologic origin (6, 7).

The incidence of type 2 diabetes in Iran is about 5.1 million people and because of this high incidence we conducted this study on this group (8). The purpose of this study was audiologicaly evaluating the diabetic patients (type 2) who referred to E.N.T clinic of Ayatollah Rouhani Hospital in Babol and identifying the amount of hearing loss in this population.

## Methods

The hearing status of 50 patients with type 2 mellitus diabetes (case group) was compared to 50 normal subjects in the control group. The two groups were matched in sex and age. In both groups, 40 women and 10 men were contributed. The mean age of control group was 49.98±3.2, and 50.10±3.02 years old for the case group. Both groups were in the age bracket of 45 to 55 years old. The case group was selected from diabetic patients in Ayatollah Rouhani Hospital with diabetes for 10 years or more, and the control group was composed of individuals with no history of diabetes selected randomly. 

Two questionnaires were filled out. Questionnaire number ''1'' was for the diabetics that include information about the history and duration of this disease and questionnaire number 2 was for both groups, which asked about sex, age, history of smoking, history of ear disease or ototoxic drugs consumption.

Then, clinical otoscopy and examination were done for both groups. If they did not have any problem such as impact cerumen, external serous otitis, they were referred to the audiology clinic of Ayatollah Rouhani Teaching Hospital, in Babol. 

In the audiology clinic, P.T.A (pure- tone- audiometry) and speech tests including SRT (speech reception threshold) and SDS (speech discrimination score) were performed. P.T.A included measuring pure tone hearing thresholds for 250 Hz, 500Hz, 1000 Hz, 2000 Hz, 4000Hz, 6000Hz, 8000Hz frequencies in both ears. 

Thresholds more than 15dbHL was considered as hearing loss (9). SRT assesses the patient’s ability to respond to the standardized two syllabic words and S.D.S evaluates the patient’s ability to respond to standardized monosyllabic words (10). 

All the collected data were computed with SPSS software Version 18 and t-test statistic exam and p<0.05 was considered as a significant level.

## Results

Fifty diabetic and 50 non-diabetic patients were enrolled in this study. 40 cases of each group were females and ten of them were males. In table 1, the mean average of hearing thresholds in the different frequencies for the right and the left ears is shown.

In table 1, the hearing loss in all the frequencies was more in the diabetic group than the control one, and these differences were statistically significant in all frequencies. In table 2, the mean speech reception thresholds (SRT) and speech discrimination score (SDS) for both groups were shown. The results indicated that SRT was worse in the diabetic group than the control group and SDS was poorer in the diabetic group than the control group.

**Table1:** Mean hearing thresholds for the right ear and left ear between the diabetic and non-diabetic individuals

	**Frequencies** **(HZ)**	**Diabetic** **(db)**	**Non Diabetic** **(db)**	**Pvalue**
Right ear	250	10.55.64	6.084.2	0.0001
	500	10.76.06	7.064.94	0.001
	1000	15.27.14	8.165.02	0.0001
	2000	169.63	10.446.13	0.001
	4000	21.213.46	12.867.86	0.0001
	6000	26.3616.5	15.669.4	0.0001
	8000	28.320	17.8412.22	0.002
left ear	250	11.25.3	6.064.29	0.0001
	500	9.74.88	7.284.88	0.015
	1000	127.14	8.864.6	0.011
	2000	14.29.44	11.065.86	0.049
	4000	20.913.27	13.767.51	0.001
	6000	25.1215.6	16.368.88	0.001
	8000	27.620.18	19.0610.79	0.01

HZ: Hertz db: Decibel

**Table 2:** Comparision of mean of SRT and SDS for both ears in diabetic and non-diabetic

		**Diabetic**	**Non Diabetic**	**P value**
Right ear	SRT	15.77.69	9.24.77	0.0001
	SDS	94.44.35	96.163.32	0.025
left ear	SRT	13.56.87	9.14	0.0001
	SDS	94.323.71	95.923.18	0.023

## Discussion

In this study, we found that the diabetic patients had more hearing loss in all frequencies in both ears in comparison with the normal group. In the study of Orts et al., the etiology of hearing loss in diabetes was proposed as impairment of outer hair cell function (11). In Nageris et al.’s study, impairment in hair cells in pathogenesis of hearing loss in diabetes was rejected (12).

These differences were more considerable in the right ear, but there was not any considerable differences among different frequencies. These results are consistent with the study results of Cullen et al. and Panchu et al. (13, 14). In the study of Safavi et al. the diabetic patients had more hearing loss in high frequencies (4,8khz) compared to the non -diabetic patients and this loss was more in the chronic diabetic patients (15).

In this study, we found that SRT was worse in the diabetic group in comparison with the control group and SDS in diabetic group was worse than the control group. This result is consistent with Frisina et al.’s study (16). The rate of hearing loss in diabetic patients was more in all frequencies in both ears in comparison with the control group, but in cases like presbycusis and acoustic traumas, hearing loss exits at high frequencies.

Thus, when every middle-aged patient with mild to moderate sensory-neural. Hearing loss in all frequencies refers to E.N.T audiology clinic, one probable diagnosis that should be considered is that hearing loss is caused by mellitus diabetes. The weakness of this study may be the duration of disease that we have not considered in this study. Another weakness of this study may be the lack of facility for performing of HbA1C. Other studies with the consideration of these variables are urgently recommended. In conclusion, the results show that hearing loss in different frequencies and speech reception thresholds were higher in the diabetic group but speech discrimination score was higher in the normal individuals. Audiometry and speech audiometry monitoring are recommended in all diabetic patients during therapy.

## Notes

**Funding: **This study was supported by Babol University of Medical Sciences, a part thesis of Pouria Taghinejad Omran.

## Notes

**Ethical approval: **This study obtained ethics committee approval.
